# Enhanced asthma-related fibroblast to myofibroblast transition is the result of profibrotic TGF-β/Smad2/3 pathway intensification and antifibrotic TGF-β/Smad1/5/(8)9 pathway impairment

**DOI:** 10.1038/s41598-020-73473-7

**Published:** 2020-10-05

**Authors:** Dawid Wnuk, Milena Paw, Karolina Ryczek, Grażyna Bochenek, Krzysztof Sładek, Zbigniew Madeja, Marta Michalik

**Affiliations:** 1 Department of Cell Biology, Faculty of Biochemistry, Biophysics and Biotechnology, Jagiellonian University, Gronostajowa 7, 30-378 Kraków, Poland; 2 Department of Internal Medicine, Faculty of Medicine, Jagiellonian University Medical College, Krakow, Poland

**Keywords:** Respiratory system models, Growth factor signalling, Asthma

## Abstract

Airway remodelling with subepithelial fibrosis, which abolishes the physiological functions of the bronchial wall, is a major issue in bronchial asthma. Human bronchial fibroblasts (HBFs) derived from patients diagnosed with asthma display in vitro predestination towards TGF-β_1_-induced fibroblast-to-myofibroblast transition (FMT), a key event in subepithelial fibrosis. As commonly used anti-asthmatic drugs do not reverse the structural changes of the airways, and the molecular mechanism of enhanced asthma-related TGF-β_1_-induced FMT is poorly understood, we investigated the balance between the profibrotic TGF-β/Smad2/3 and the antifibrotic TGF-β/Smad1/5/9 signalling pathways and its role in the myofibroblast formation of HBF populations derived from asthmatic and non-asthmatic donors. Our findings showed for the first time that TGF-β-induced activation of the profibrotic Smad2/3 signalling pathway was enhanced, but the activation of the antifibrotic Smad1/5/(8)9 pathway by TGF-β_1_ was significantly diminished in fibroblasts from asthmatic donors compared to those from their healthy counterparts. The impairment of the antifibrotic TGF-β/Smad1/5/(8)9 pathway in HBFs derived from asthmatic donors was correlated with enhanced FMT. Furthermore, we showed that Smad1 silencing in HBFs from non-asthmatic donors increased the FMT potential in these cells. Additionally, we demonstrated that activation of antifibrotic Smad signalling via BMP7 or isoliquiritigenin [a small-molecule activator of the TGF-β/Smad1/5/(8)9 pathway] administration prevents FMT in HBFs from asthmatic donors through downregulation of profibrotic genes, e.g., α-SMA and fibronectin. Our data suggest that influencing the balance between the antifibrotic and profibrotic TGF-β/Smad signalling pathways using BMP7-mimetic compounds presents an unprecedented opportunity to inhibit subepithelial fibrosis during airway remodelling in asthma.

## Introduction

The airway tracts of patients diagnosed with asthma are characterized by chronic inflammation along with remodelling of the bronchial wall^1, 2^. This phenomenon is a complex process involving damage to the epithelial layer, hypertrophy and hyperplasia of the smooth muscles, enhanced vasculature and the key phenomenon subepithelial fibrosis^3^. The progression of the latter is linked with the expansion of myofibroblasts characterized by the high contractile apparatus and secretory hyperactivity of bronchial wall cells for i.e. the overproduction of extracellular matrix proteins^4, 5^. Although, in last years, it was shown the multiple origin of myofibroblasts^6, 7^, recent lineage tracing studies confirm that the main sources of myofibroblasts in bronchial wall are resident mesenchymal cells such as fibroblasts and pericytes^8–11^. In 2009, we were the first to show that the local resident bronchial fibroblasts isolated from biopsies derived from asthmatic patients had a substantially higher potential for transforming growth factor β (TGF-β)-induced fibroblast to myofibroblast transition (FMT) than those from their non-asthmatic counterparts^12^. These cells displayed some inherent features that facilitate their differentiation into myofibroblasts^4, 13, 14^.

In our previous studies, we showed that the architecture of the actin cytoskeleton, which was associated with N-cadherin function^14, 15^, Cx43 signalling activity^16^ and the functional status of TGF-β_1_ and connective tissue growth factor (CTGF)^17, 18^ as the main stimulators of FMT through the Smad2/3 signalling pathway, participated in the efficient myofibroblast transition increase in fibroblasts from asthmatic patients^12, 16^. However, to date, the reason why fibroblasts from asthmatic patients react more strongly to TGF-β_1_ and, as a result, show higher transformation to myofibroblasts than those of their non-asthmatic counterparts is unclear.

The importance of TGF-β_1_ signalling in asthma pathogenesis and its duration has been illustrated by genome-wide association studies^19, 20^ and many physiological and functional studies^21, 22^. TGF-β_1_, which is mainly secreted by bronchial epithelial cells and eosinophils, is overproduced in bronchoalveolar lavage fluid (BALF)^23^ and in many lung submucosal cells from patients with asthma. Its profibrogenic activity in asthma is shown by the intensification of the immune response and bronchial remodelling in different layers of the bronchial wall, including subepithelial fibrosis^22^.

Cells exposed to TGF-β_1_ show activation of type II serine-threonine kinase TGF-β receptor (TGF-β-RII), which induces phosphorylation of activin receptor-like kinases (ALKs)^24–27^. ALK5 (also known as TGF-βRI) is a typical ALK activated after TGF-β_1_ treatment and transmits phosphorylation on regulatory Smad proteins (R-Smads) through the Smad2/3 pathway after activation. Although TGF-β_1_ is believed to induce Smad2/3 phosphorylation, some studies have shown that it can also induce Smad1/5/(8)9 phosphorylation, which is mainly dependent on ALK1 or ALK2 activity^26^. Phospho-R-Smads (p-Smad2/3 or p-Smad1/5/9) oligomerize with common Smads (Co-Smad; Smad4) and then translocate into the cell nucleus, where they bind gene promoters/enhancers and activate or repress the transcription of target genes^28^. Inhibitory Smad proteins (I-Smads; Smad 6 and 7) and Smad ubiquitin regulatory factors (Smurfs: Smurf 1, Smurf 2 and Arkadia) play a significant role in the regulation of signal transduction through TGF-β/Smad-signalling pathway. Smad7 prevents the activation of TGF-β1 signalling by competitive inhibition of the interaction of R-Smads with TGF-βRI. Smurf 1 and Smurf 2 (E3 ubiquitin ligases) play a crucial role in the recognition and ubiquitin-dependent degradation of TGF-β receptors and regulate the basal level of both R Smads (Smad 2, Smad 3 and Smad 1), and I-Smads (Smad 7) by targeting them for degradation28. It was shown that in fibroblasts, Smurf 2 preferentially targets Smad1 for ubiquitination and proteasome-mediated degradation.This protein has a much weaker effect on Smad2 levels, but does not affect Smad3 levels^29^.

Smad2/3 signalling is important for extracellular TGF-β_1_ stimuli. Smad2/3 pathway activation was reported to induce a profibrotic response^30–33^, whereas Smad1/5/9 activation plays an antifibrotic role in different tissues^34, 35^. The Smad1/5/9-regulated pathway is activated mainly by bone morphogenic protein receptors (R-BMPs)^24^. Although our recent reports indicated the involvement of TGF-β/Smad2/3 signalling in FMT efficiency, whether the enhanced TGF-β_1_-induced FMT potential in human bronchial fibroblast (HBF) populations derived from asthmatic donors compared to that of non-asthmatic donors may be the result of differential regulation of Smad signalling is still unclear^16, 36–38^. Thus, elucidation of the cellular/molecular mechanisms by which TGF-β_1_ favours myofibroblast accumulation during subepithelial fibrosis in asthma remodelling progression remains the aim of many research groups. However, a comparison of the activity of the profibrotic Smad2/3 pathway and the antifibrotic Smad1/5/9 pathway has never been conducted. To further elucidate the molecular mechanisms of FMT based on TGF-β/Smad signalling in asthma, we used an in vitro model of cultured HBFs isolated from ex vivo bronchial biopsies of patients with diagnosed asthma (AS) and donors in whom asthma was excluded during clinical diagnosis (NA).

The aim of the present study was to evaluate whether enhanced asthma-related TGF-β_1_ FMT is the result of the differential activation of the profibrotic TGF-β/Smad2/3 and antifibrotic TGF-β/Smad1/5/9 signalling pathways in HBFs.

## Results

### Enhanced FMT efficiency in HBFs from asthmatic donors is due to the increased activation of the profibrotic Smad2/3 pathway

**Figure 1:**
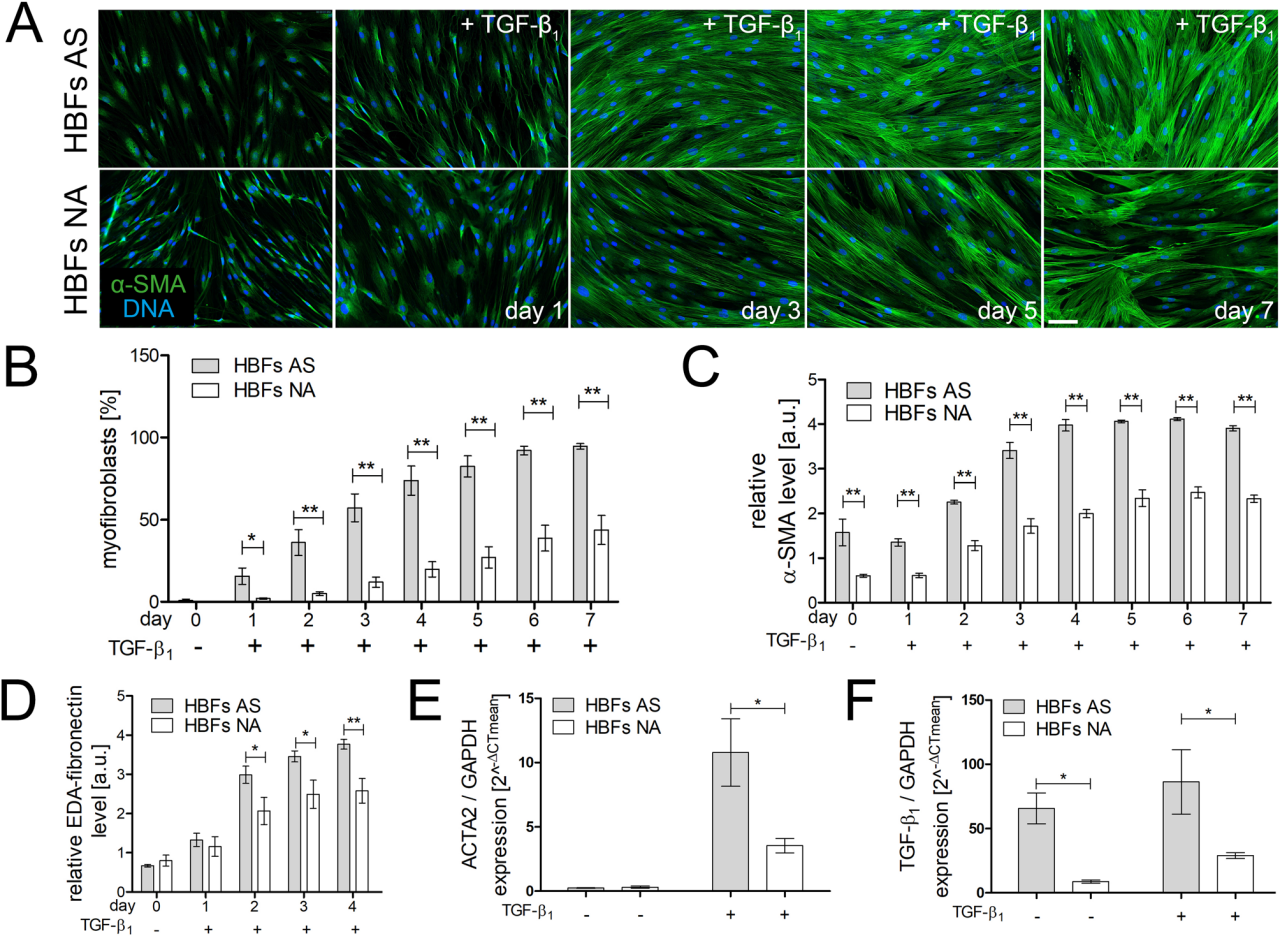
HBFs derived from patients with asthma transdifferentiate into myofibroblasts efficiently and faster than those of their healthy counterparts. HBFs (AS = 10; NA = 6) were cultured in serum-free conditions in the absence or presence of TGF-β_1_ (5 ng/mL) for 0–7 days. **(A)** Then, the cells were fixed with 3.7% formaldehyde, permeabilized, and immunostained for α-SMA (green) and counterstained for DNA (blue), as shown on representative images. Scale bar = 25 μm. **(B)** The fraction of cells with prominent α-SMA-positive stress fibres in HBF populations was determined using fluorescence microscopy, each in three independent experiments. **(C,D)** Main markers of myofibroblast formation: α-SMA and EDA-fibronectin levels were assessed using *in-cell-ELISA* tests, and the results are presented as the mean value of absorbance (450 nm) reflecting the protein content. Data represent the mean ± SEM carried out on HBFs (AS = 10; NA = 6), each in triplicate. (**E,F**) HBFs (AS = 7; NA = 7) were cultured in serum-free conditions in the absence or presence of TGF-β_1_ for 24 h. RT-qPCR analyses of alpha smooth muscle actin (*ACTA2*) and *TGF-β*_*1*_ expression were performed. Statistical significances of all experiments were tested using the non-parametric Mann–Whitney test; *p ≤ 0.05, **p ≤ 0.01.

As mentioned above, TGF-β_1_-induced FMT was intensified in vitro in HBFs from asthmatic patients^12, 15, 16^. Immunofluorescence analyses of α-smooth muscle actin (α-SMA), the main marker of myofibroblasts, showed that FMT also occurs much more rapidly in fibroblasts from patients with diagnosed asthma than those from non-asthmatic donors after exposure to TGF-β_1_ (Fig. 1A,B). These data were also confirmed by RT-qPCR and ELISA analyses. The results revealed that HBFs from asthmatic donors displayed significantly higher gene expression of α-SMA and extra domain A of fibronectin (EDA-fibronectin), especially after TGF-β_1_ stimulation, compared with those from their non-asthmatic counterparts (Fig. 1C–E). Moreover, the gene expression analyses of the profibrotic factor TGF-β_1_ showed that it was correlated with FMT efficiency in the HBF populations of both groups (Fig. 1F comp. Fig. 1E and Fig. S1A).

**Figure 2:**
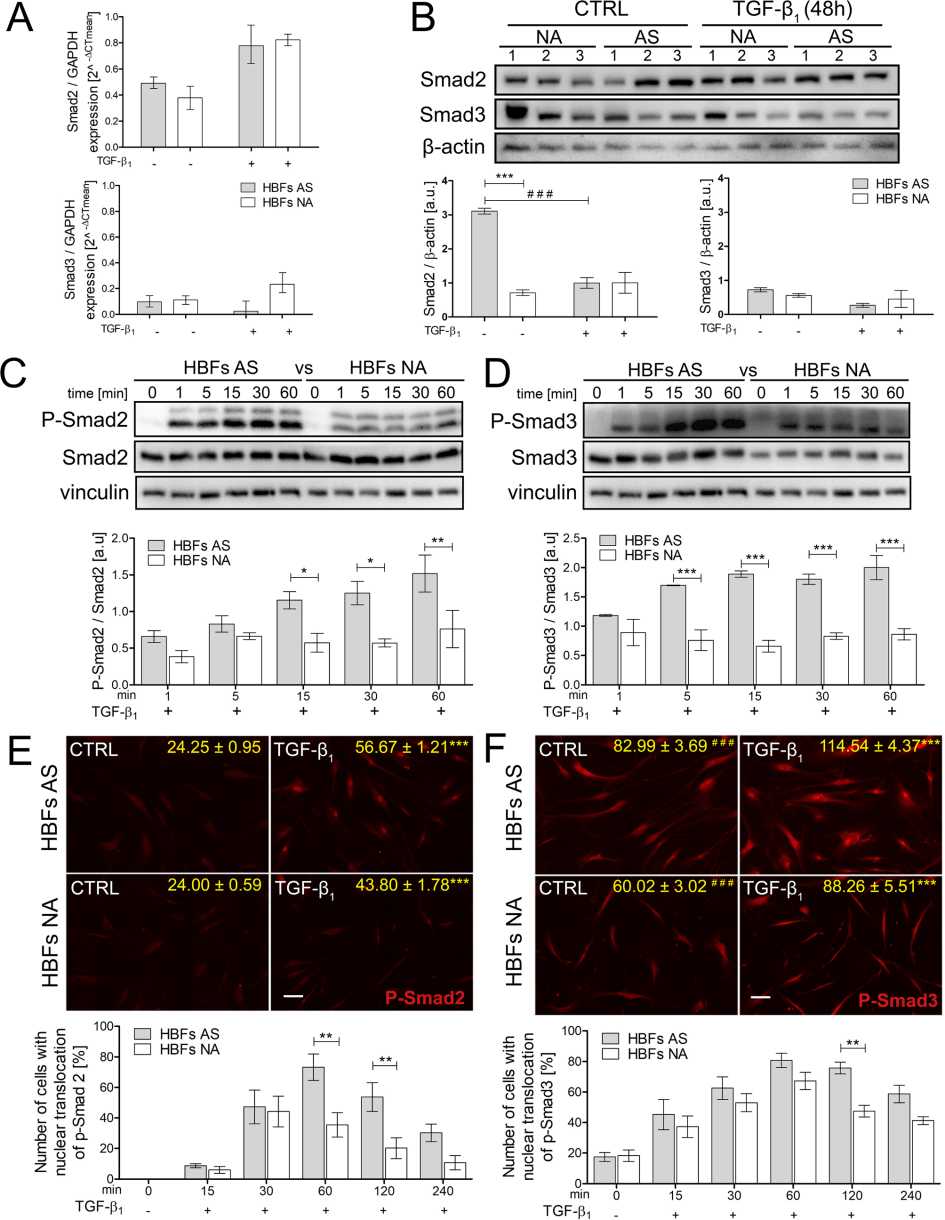
The TGF-β_1_/Smad2/3 pathway is intensified in HBFs derived from patients with asthma compared to their healthy counterparts. Cells (AS = 7, NA = 7) were cultured in serum-free medium with or without TGF-β_1_ (5 ng/mL) for 24 h (**A**), 48 h (**B**), 1–60 min (**C**,**D**) or 0–240 min (**E**,**F**). **(A)** Smad expression was analysed at the mRNA level using RT-qPCR. **(B-D)** Smad2, Smad3, and their phosphorylated forms were detected using Western blots. Representative membranes are shown. Densitometric quantification of Smad proteins in relation to β-actin and p-Smad2 or 3 in relation to appropriate Smads (as control proteins) are presented on the graphs as values of relative optical densities (ROD) (n = 6). **(E**–**F)** HBFs were fixed, permeabilized, and immunostained for p-Smad2 or p-Smad3. Representative photos were selected. Scale bar = 25 μm. The percentage of cells with p-Smad + nuclei was determined using fluorescence microscopy. Alternatively, p-Smad levels in HBF nuclei were quantified with the fluorometric approach using ImageJ, and the results are presented as the mean fluorescence intensity in relation to the nuclei area. Data on photos (yellow) represent the mean ± SEM of AS = 5, NA = 5; each in min. 100 cells. Statistical significance (**C**–**F**) * between HBFs AS TGF vs HBFs NA TGF; (**F**) ^#^ HBFs AS CTRL vs HBFs NA CTRL. Statistical significance was tested using the non-parametric Mann–Whitney test; * *p* ≤ 0.05, **p ≤ 0.01, *** *p* ≤ 0.001.

The canonical TGF-β_1_/Smad2/3 signalling pathway is crucial for FMT^21, 22^. Since we previously observed enhanced TGF-β_1_-induced FMT in HBFs from asthmatic donors, we further focused on analysis of Smad2 and Smad3 expression (at the mRNA and protein levels) between HBFs derived from asthmatic and non-asthmatic donors. RT-qPCR analyses confirmed that there were no significant differences in Smad2 and Smad3 mRNA levels between HBFs from asthmatic and non-asthmatic donors in control conditions and after 24 h of stimulation with TGF-β_1_ (Fig. 2A). Moreover, Smad2 protein content detected by immunoblots was increased in unstimulated HBFs from asthmatic donors compared to non-asthmatic donors, but TGF-β_1_ notably decreased Smad2 and Smad3 levels in the HBF populations from both studied groups (Fig. 2B). Interestingly, the TGF-β_1_-induced phosphorylation of Smad2 and Smad3 was significantly stronger in HBFs from asthmatic donors (Fig. 2C,D) than those from non-asthmatic donors. Differences in Smad3 phosphorylation were observed after just 5 min and were more pronounced than those of Smad2 phosphorylation, which were significant after 15 min of TGF-β_1_ stimulation (Fig. 2C,D). Smad2/3 pathway activation resulted in increased nuclear localization of p-Smad2/3. The nuclear translocation of activated Smad complexes, verified by immunofluorescence, was the strongest after 60 min of TGF-β_1_ stimulation and further enhanced after 120 min between the studied groups (Fig. 2E,F). Moreover, the mean nuclear fluorescence intensity of p-Smad3 was significantly different between AS and NA HBFs under control conditions (Fig. 2F).

### The antifibrotic Smad1/5/(8)9-dependent pathway is overactivated in HBFs isolated from non-asthmatic donors

**Figure 3:**
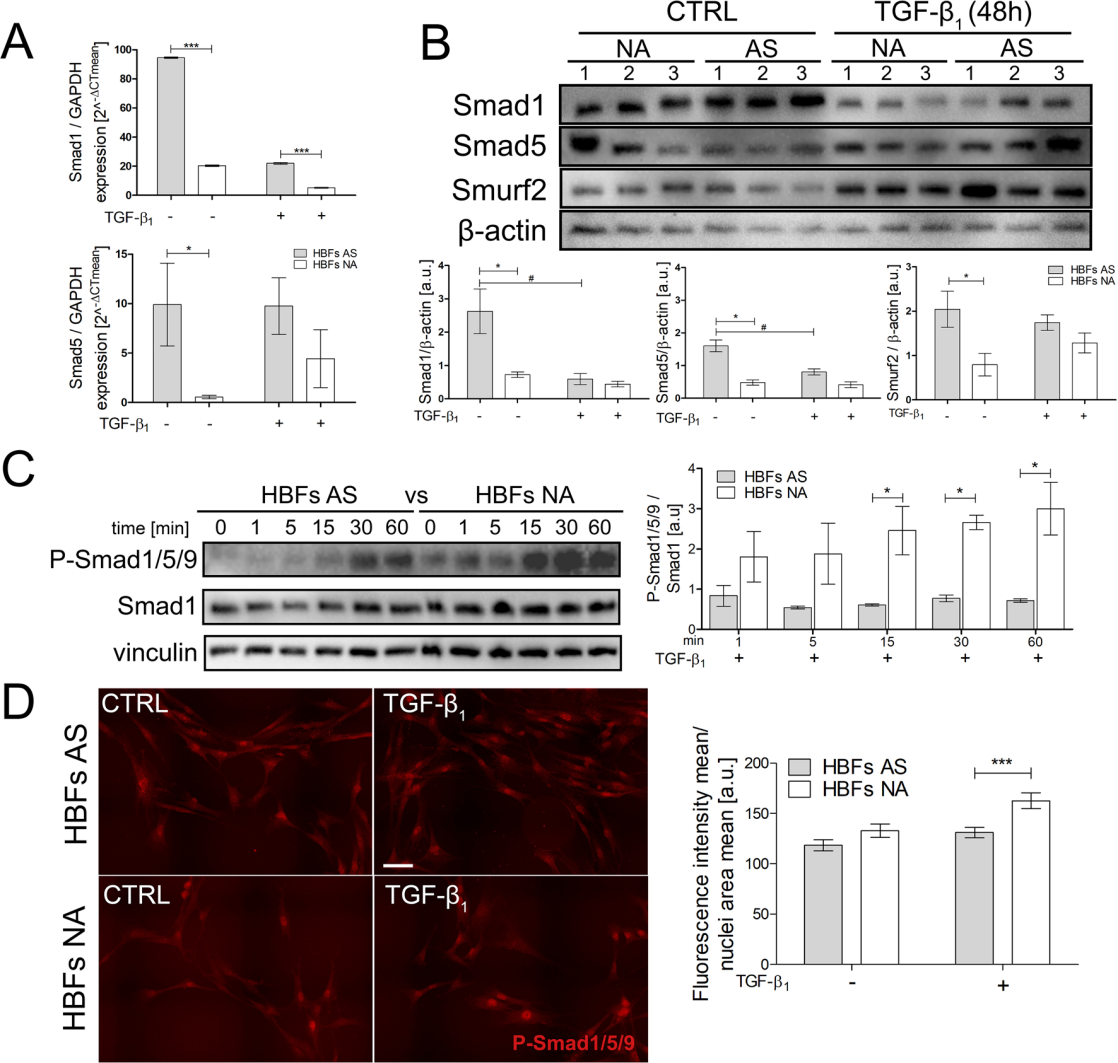
The TGF-β_1_/Smad1/5/9 pathway is intensified in HBFs derived from patients without asthma compared to asthmatic patients. **(A)** Cells were cultured in serum-free medium supplemented with TGF-β_1_ (5 ng/mL) for 24 h. Then, the mRNA was isolated, and transcripts were analysed using RT-qPCR. **(B)** Smad1, Smad5, and Smurf2 were detected using Western blots. Representative membranes are shown. Densitometric quantification is presented on the graphs as values of the relative optical densities (ROD) (n = 6) of the bands in relation to that of β-actin (control protein). **(C)** Phosphorylated forms of Smad1/5/9 (p-Smad1/5/9) were detected using Western blots. Representative membranes are shown. Densitometric quantification is presented on the graph as values of the relative optical densities (ROD) (n = 4) of p-Smads in relation to Smad1 (as the control protein) **(D)** HBFs cultured in serum-free medium were fixed, permeabilized, and immunostained for p-Smad1/5/9, and the percentage of cells with p-Smad1/5/9-positive nuclei was determined using fluorescence microscopy. Representative photos were selected. Scale bar = 25 μm. The mean fluorescence intensity in relation to the nuclei area (AS = 3, NA = 3 each in 200 cells) was measured using ImageJ. Statistical significance was tested using the non-parametric Mann–Whitney test; *p ≤ 0.05, ***p ≤ 0.001 or the T-test; ^#^*p* ≤ 0.05.

Because some reports have shown that the Smad1/5/(8)9 pathway plays a crucial antifibrotic role in renal and liver^39–41^, further analyses were performed to assess TGF-β_1_/Smad1/5/9 pathway activity in the HBF populations from asthmatic and non-asthmatic donors. RT-qPCR studies showed that the Smad1 and Smad5 levels were fivefold and tenfold higher, respectively, in HBFs derived from AS donors (Fig. 3A) than those from non-asthmatic donors. Interestingly, TGF-β_1_ stimulation strongly suppressed Smad1 expression, which was similar in the fibroblasts derived from both study groups. The effect of TGF-β_1_ on Smad5 expression was undetectable (Fig. 3A). Parallel immunoblot analyses revealed differences between the Smads at the protein level in the HBFs from both study groups (approximately threefold increases in both the Smad1 and Smad5 levels in unstimulated HBFs from asthmatic donors) (Fig. 3B). However, TGF-β_1_ stimulation strongly decreased Smad1 but not Smad5 contents in HBFs from patients with asthma. The level of the E3 ubiquitin-protein ligase Smurf2 was also increased in AS HBFs. A comparison of the Smad1/5/9 pathway activity between HBFs derived from asthmatic and the non-asthmatic donors was performed by immunoblots and immunofluorescence analysis. A representative immunoblot membrane showed a time-dependent increase in the phosphorylation of Smad1/5/9 after TGF-β_1_ treatment, resulting in faster and stronger (three times) phosphorylation of Smad1/5/9 in the HBFs from non-asthmatic donors than those from asthmatic donors (Fig. 3C). Moreover, the nuclear localization of phosphorylated Smad1/5/9 complexes, shown on representative images and measured fluorometrically, was also enhanced in HBFs from non-asthmatic donors after TGF-β_1_ treatment (Fig. 3D). Since it is well documented that phosphorylated R-Smads translocate into nucleus in complexes with Co-Smads we compared the expression of Smad 4 at the mRNA and protein levels in the HBFs from both study groups. Our results of RT-qPCR analyses showed significantly higher Smad 4 expression in HBF populations derived from asthmatics (Fig. S1D). Interestingly, immunoblot analyses revealed no differences between Smad 4 at the protein level in the HBF populations from AS and NA donors (Fig. S1E).

### Silencing of Smad1 in the HBFs derived from non-asthmatic donors leads to Smad2/3 pathway stimulation and FMT activation

**Figure 4:**
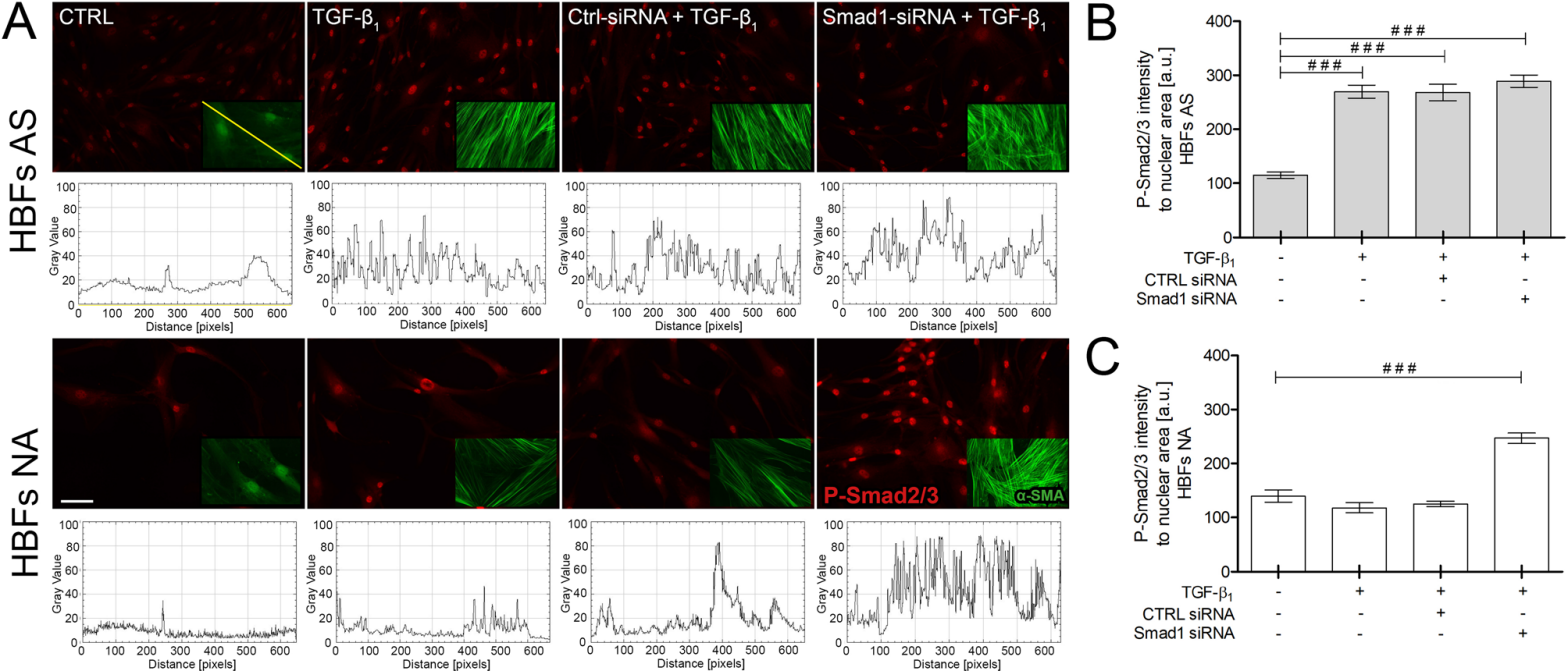
The Smad2/3 pathway is overactivated in HBFs derived from patients without asthma after Smad1 silencing. **(A)** HBFs cultured in serum-free medium were transfected with siRNA-Smad1 or control-siRNA, treated with TGF-β_1_ (5 ng/ml) for 1 h (photos) and 5 days (inserts), fixed, permeabilized, and immunostained for p-Smad2/3 (red) and α-SMA (green), as shown on representative images and inserts. The fluorescence intensity of α-SMA-enriched fibres is presented on the plot profiles (under the photos). **(B,C)** Fluorescence intensity of p-Smad2/3-positive nuclei was determined using fluorescence microscopy at the same excitation and analysed by ImageJ. Representative photos were selected. Scale bar = 100 μm. Data represent the mean ± SEM of 100 cells (AS = 3, NA = 3); each in duplicate. Statistical significance was tested using one-way ANOVA with the Bonferroni multiple comparison post hoc test; ^###^*p* ≤ 0.001.

To illustrate the impact of the opposing Smad-dependent pathways on FMT, we silenced the Smad1 gene in HBFs. The immunofluorescence staining of p-Smad2/3 proteins after 1 h of TGF-β_1_ administration showed that Smad1 silencing had no impact on Smad2/3 pathway activation in the HBFs from asthmatic donors, but it caused a twofold increase in p-Smad2/3 activity (enhanced nuclear translocation of p-Smad2/3 complexes) in the HBFs from non-asthmatic donors (Fig. 4A). This observation was confirmed by fluorometric analysis (Fig. 4B,C). This change resulted in the intensification of the FMT process (Fig. 4A). The immunofluorescence analyses showed that stress fibres consisting of α-SMA were increased after Smad1 silencing in the HBF populations from non-asthmatic donors (Fig. 4A, inserts with plots).

### Activation of Smad1/5/(8)9 signalling prevents FMT in HBFs from asthmatics

**Figure 5:**
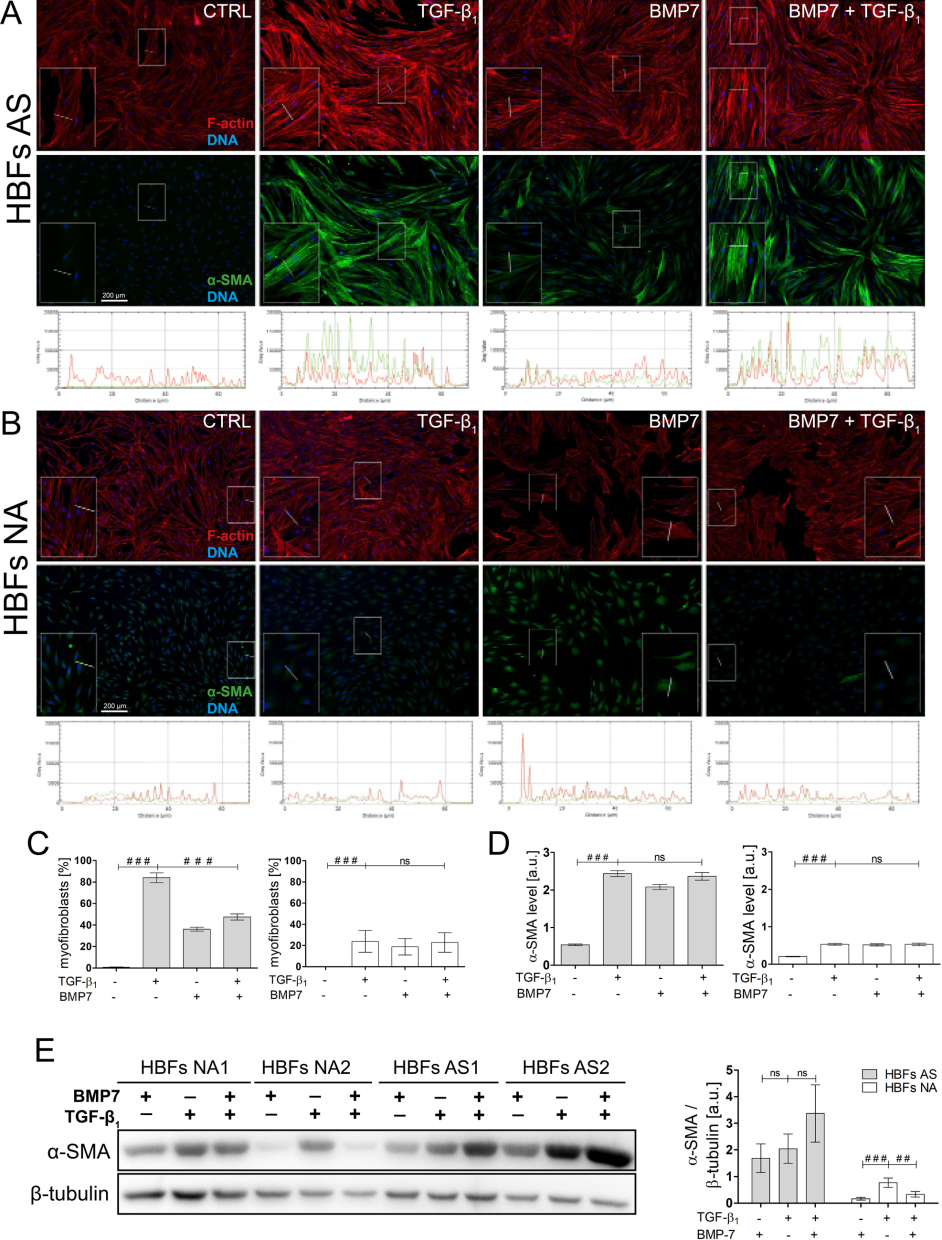
Smad2/3 *versus* Smad1/5/9 activation balance plays a pivotal role in fibroblast to myofibroblast transition of HBFs. **(A,B)** HBFs were cultured in serum-free medium without (Ctrl) or with TGF-β_1_ (5 ng/ml) in the absence or presence of BMP7 (100 ng/ml) for 7 days. Then, the cells were fixed, permeabilized, and immunostained for α-SMA (green) and counterstained for F-actin (red) and DNA (blue), as shown on representative images. The intensity of actin fibre fluorescence in the sections is presented on plot profiles and quantified in graphs (under the photos). Scale bar = 200 μm. **(C,E)** The fraction of cells with prominent α-SMA-positive stress fibres in HBF populations was determined using fluorescence microscopy in three independent experiments. **(D,F)** Analyses of α-SMA content were carried out using an *in-cell* ELISA test, and the results are presented as the mean value of absorbance (450 nm) reflecting the protein content. Data represent the mean ± SEM carried out on HBFs (AS = 10; NA = 6), each in triplicate. **(G)** α-SMA was detected using Western blots. Representative membranes are shown. Glyceraldehyde 3 phosphate dehydrogenase (GAPDH) was used as a loading control. **(H)** Densitometric quantification of membranes is presented on the graph as values of the relative optical densities (ROD) (n = 2) of α-SMA in relation to GAPDH (as control protein) **(A,C,D)** HBFs from asthmatics, **(B,E,F)** HBFs from non-asthmatics. Statistical significance was tested using were determined using Statistical significance was tested using one-way ANOVA with the Bonferroni multiple comparison post hoc test; ns – non statistically significant, ^#^*p* ≤ 0.05, ^##^*p* ≤ 0.01, ^##*#*^*p* ≤ 0.001.

We used BMP7, which is described as a main activator of the Smad1/5/(8)9 pathway^42–44^, to assess the impact of Smad1/5/9 activation on the FMT process in HBF populations. Immunofluorescence analyses showed that BMP7 stimulates the myofibroblast transition of HBFs from asthmatic donors, but this process was much weak than after TGF-β_1_ administration (Fig. 5A,B,C). These microscopic observations were confirmed by fluorometric analyses, which revealed shorter, thinner and less enriched α-SMA actin stress fibres in BMP7-treated HBFs compared to TGF-β_1_-stimulated HBFs (Fig. 5A,B). The combination of BMP7 and TGF-β_1_ decreased the FMT potential (measured as the number of myofibroblasts) in the HBFs derived from asthmatic patients but not in those from their non-asthmatic counterparts (Fig. 5C). However, α-SMA levels were not reduced after TGF-β_1_ and BMP7 treatment in the HBFs derived from asthmatic donors (Fig. 5D,E). These results suggested that BMP exerts an inhibitory effect on the TGF-β_1_-induced FMT by affecting the actin cytoskeleton architecture.

**Figure 6:**
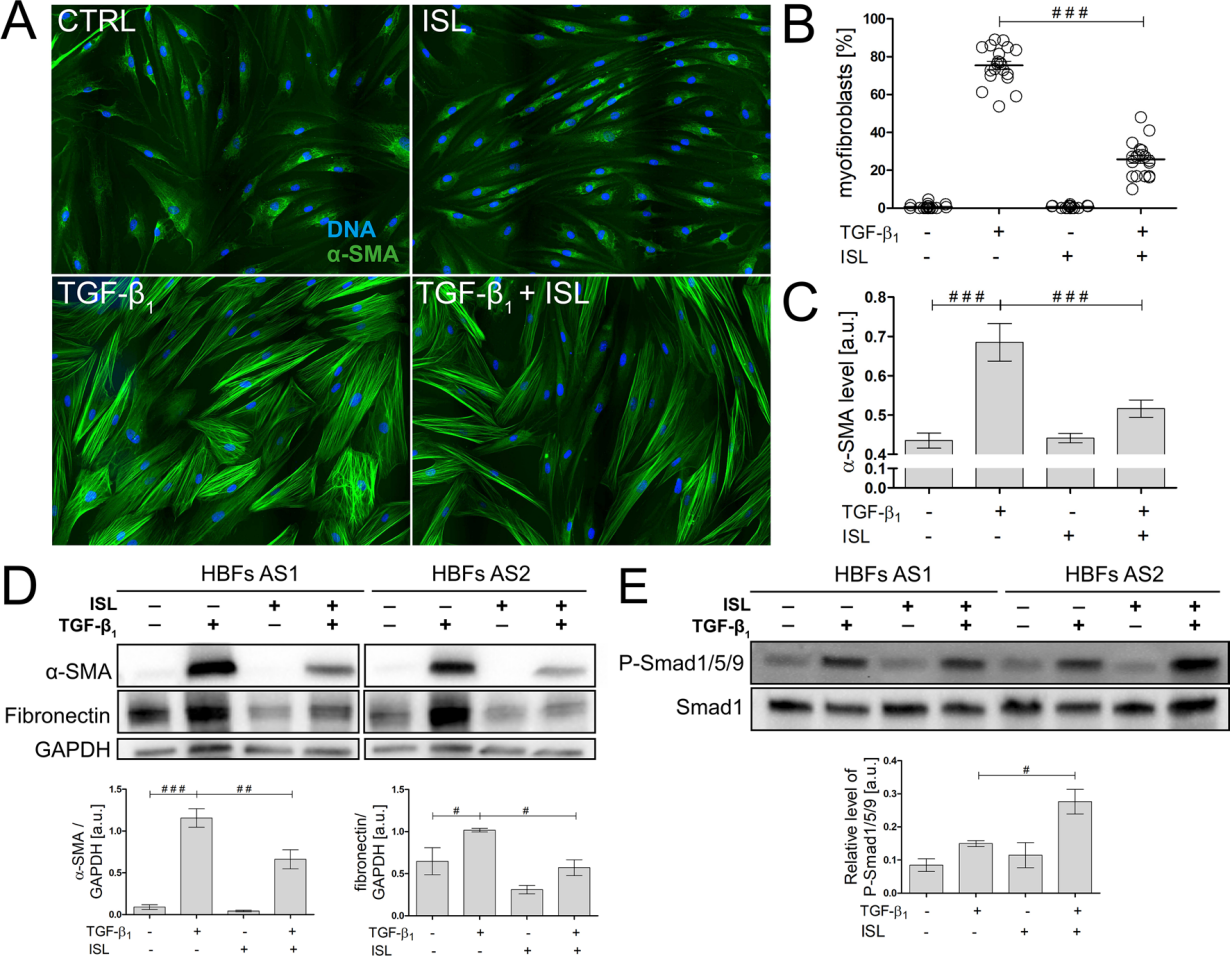
Isoliquiritigenin (ISL) attenuates the TGF-β_1_-induced phenotypic transition of HBFs into myofibroblasts through Smad1/5/9 pathway stimulation. **(A)** HBFs derived from asthmatic donors were cultured in serum-free medium supplemented with TGF-β_1_ (5 ng/mL) or not (Ctrl) in the absence or presence of ISL (25 μM) for four days. Then, the cells were fixed with 3.7% formaldehyde, permeabilized, and immunostained for α-SMA (green) and DNA (blue), as shown on representative images. Scale bar = 25 μm. **(B)** The fraction of cells with prominent α-SMA^+^ stress fibres (myofibroblasts) in HBF populations (n = 6) was determined using fluorescence microscopy in three independent experiments. **(C)** α-SMA content was defined using *in-cell* ELISA, and the results are presented as the mean value of absorbance (450 nm) reflecting the protein content. Data represent the mean ± SEM carried out on HBFs AS (n = 6), each in duplicate. **(D)** Analyses of α-SMA and fibronectin content were carried out in total cell lysates from HBFs cultured as in (A) using immunoblotting. Glyceraldehyde 3 phosphate dehydrogenase (GAPDH) was used as a loading control. The effect of ISL on the α-SMA levels in the TGF-β_1_-treated HBFs is presented as a bar graph and shows densitometric quantification of the Western blots. Data are the mean ± SEM (n = 5). **(E)** The activation of the Smad1/5/9 pathway was determined using Western blot analysis of Smad1/5/9 phosphorylation in relation to Smad1 and GAPDH as a loading control. Statistical significance was tested using the t-test; ^#^*p* ≤ 0.05, ^##^*p* ≤ 0.01, ^##*#*^*p* ≤ 0.001.

Further analyses were performed to estimate the effect of isoliquiritigenin (ISL), described as a small molecule activator of BMP signalling^45^, on the TGF-β_1_-induced FMT in the HBF populations derived from asthmatic patients. ISL (used at a concentration that did not affect cell viability and proliferation, Fig. S1F,G) attenuated the TGF-β_1_-induced phenotypic differentiation of HBFs, as shown by a reduction in the fraction of myofibroblasts in the HBF populations (Fig. 6A,B). Concomitantly, ISL decreased the α-SMA and fibronectin levels in TGF-β_1_-treated HBFs (Fig. 6C,D). Moreover, immunoblot analyses confirmed that ISL-induced FMT attenuation is dependent on Smad1/5/9 pathway overactivation (Fig. 6E).

## Discussion

A major problem of bronchial remodelling in asthma is progressive subepithelial fibrosis, which causes irreversible changes in the bronchial wall^1, 2, 5, 46^. The strong accumulation of contractible (overexpression of α-SMA) myofibroblasts secreting excessive amounts of extracellular matrix proteins (fibronectin, collagens, proteoglycans) abolishes the physiological functions of the bronchi. FMT, a crucial event during subepithelial fibrosis observed in asthma, depends on the combined action of inflammatory mechanisms and inherent features of airway wall fibroblasts facilitating their phenotypic transitions in response to humoural factors^4^. The induction of the FMT in HBF populations in vitro by the addition of exogenous TGF-β_1_ mimics the in vivo asthmatic process triggered by local secretion of TGF-β_1_ (the most important pro-fibrotic cytokine^22^) within the inflammatory milieu. Therefore, the experimental model, based on the primary HBFs expanded directly from bronchial biopsies of different patients with or without diagnosed asthma, is suitable for the analyses of the FMT background in asthma, as well as for pharmacokinetic studies. Our model may have some limitations related to potential confounders such as a younger age and steroid use in the asthmatic patients in comparison to control subjects. However, due to propagation of HBFs for several passages in a culture, in our opinion, this model gives a possibility to describe the differences between cells isolated from individual patients, which are related to genetic and epigenetic background rather than to a chronic inflammation or to consequences of drugs taken by the patients. When it comes to the age differences between the both groups of patients used in our study they result from difficulties in recruiting individuals to the control group, from which we could have obtained consent and take samples from the lower respiratory tract by bronchoscopy. Although aging is recognized as a major risk factor for fibrotic diseases^47^ and FMT has been shown in in vitro studies to strongly increase with age^48^, in our studies a younger age of the asthmatic patients, in comparison to the control subjects, did not affect the enhanced TGF-β_1_-induced asthma-related FMT.

**Figure 7:**
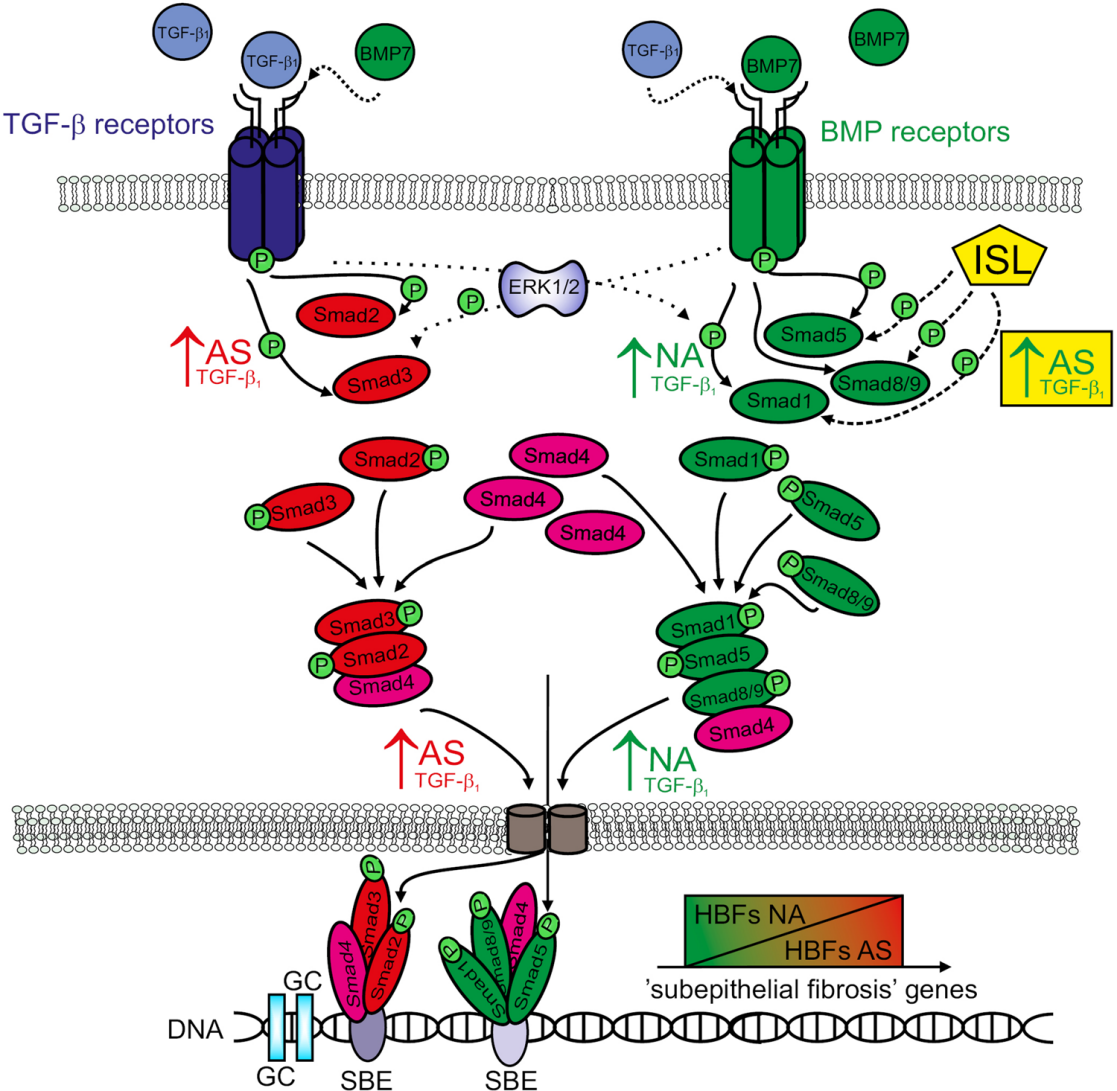
Schematic representation of interrelations between profibrotic Smad2/3-dependent and anti-fibrotic Smad1/5/(8)9-dependent pathways in HBFs. TGF-β_1_-activated profibrotic Smad2/3 pathway is enhanced in HBFs derived from asthmatic patients (↑AS; red) than in its non-asthmatic (NA) counterparts (both at the level of phosphorylation of Smad2/3 proteins and their intranuclear translocation). However, in response to TGF-β_1_ exposition, HBFs NA shows the increased activation of anti-fibrotic Smad1/5/(8)9 pathway (↑NA; green) (both at the level of phosphorylation of Smad1/5/(8)9 proteins and their intranuclear translocation). Isoliquiritigenin (ISL), is able to increasing the activation of Smad1/5/(8)9-dependent pathway (↑AS; green) and concomitant attenuation of pro-fibrotic potential in HBFs AS. Dysregulation of balance between profibrotic Smad2/3 and anti-fibrotic Smad1/5/(8)9 pathway may leads to the enhanced fibrogenic potential of asthmatic HBFs (↑AS; red). ↑—activation of pro-fibrotic pathway (red); ↑—activation of anti-fibrotic pathway (green); Abbreviations: *TGF-β*_*1*_ transforming growth factor-β_1_; *BMP7* bone morphogenic protein 7; *ISL* isoliquiritigenin; *SBE* Smad binding elements; *HBFs* human bronchial fibroblasts; *AS* asthmatic; *NA* non-asthmatic; *P* phosphorylation sites; *ERK1/2* extracellular signal-related kinase 1/2.

The FMT process is inextricably linked to profibrotic signalling from TGF-β receptors through a TGF-β/Smad2/3-dependent pathway. Our previous^12, 15, 16^ and current observations indicate that FMT is more efficient in the fibroblasts from asthmatic donors after TGF-β_1_ treatment than those from their non-asthmatic counterparts. Based on many reports describing the antifibrotic role of the Smad1/5/9 pathway in various diseases^35, 49, 50^, we compared profibrotic Smad2/3 and antifibrotic Smad1/5/9 signalling activity between the HBFs from asthmatic and non-asthmatic donors. The association of TGF-β/Smad2 signalling activity with airway remodelling in asthma has been poorly investigated^51^. We are the first to demonstrate that the intensification of the fibrogenic potential of AS HBFs was associated with increased activity of the profibrotic TGF-β/Smad2/3-dependent pathway in these cells. Both the level of phosphorylation of the Smad2 and Smad3 proteins and the nuclear translocation of their complexes were increased in AS HBFs, while NA HBFs showed much weaker activation of this pathway in vitro (Fig. 7). Such an increase in the phosphorylation of Smad2 and Smad3 was previously observed in mouse lung tissues after prolonged allergen challenge (ovalbumin)^52, 53^ and in humans in acute allergen-induced remodelling during asthma^54, 55^. The observed intensification of the TGF-β/Smad2/3 pathway in AS HBFs may be related to the enhanced expression of TGF-β receptors, especially ALK5 (Fig. S1B)^56^, because the TGF-βRII level is the same in NA and AS HBFs^36, 56^. Dysregulation of the Smad2/3 pathway through blocking of TGF-β receptors (pharmacologically and using specific antibodies) was used to decrease the FMT potential and fibrosis, but this treatment may disturb the balance of normal processes in other cells of the organism^37, 49, 57^. Therefore, substances that act downstream in the TGF-β/Smad2/3 pathway, such as inhibitors of Smad2/3 phosphorylation or nuclear translocation inhibitors (SIS3, statins, fenofibrate, etc.), are continually being assessed^16, 37, 38^.

However, some studies have shown that TGF-β can induce not only Smad2/3 phosphorylation via TGF-βRII/ALK5 binding but also Smad1/5 phosphorylation dependent on different receptor complex activities (e.g., ALK1/2/3/6) in a cell type-specific manner^58, 59^. Taken together, these observations suggest that further studies are needed to determine whether TGF-β_1_ can differentially bind to heteromeric complexes comprised of varied mammalian TGF-β family type I and type II receptors and can thus activate TGF-β/Smad pathways in diverse mechanisms in HBFs from asthmatic and non-asthmatic donors.

Smad1/5/9 pathway activation has been shown previously to have potent antifibrotic effects in liver fibrosis^41^, renal fibrosis^39, 60^, pulmonary fibrosis^61–63^ and myocardial fibrosis^64^ in animals and humans. We reported that TGF-β/Smad1/5/9 pathway activity is increased in HBFs from non-asthmatic donors compared to HBFs obtained from patients with asthma. A co-Smad4 is necessary for the nuclear translocation of Smad complexes, such as p-Smad2/3/4 and p-Smad1/5/4^28^. The observed impairment of Smad1/5 phosphorylation in TGF-β-treated HBFs from asthmatic donors along with a lack of differences in Smad4 levels between NA and AS HBFs (Fig. S1D,E) indicated overactivation of the profibrotic Smad2/3 pathway in AS HBFs. Our data are consistent with the observations that the basal expression of R-BMPs and Smad1/5 pathway activation are reduced in bronchial cells from patients with mild asthma^65^. The imbalance between the Smad1/5/9 and Smad2/3 ratio in AS HBFs can intensify the production of TGF-β_1_, fibronectin, α-SMA and collagens, leading to enhanced FMT and subepithelial fibrosis. We showed that TGF-β_1_ stimulation strongly decreased the Smad1 level in AS HBFs. This change may be caused by the increased Smurf2 level, which can degrade Smad1^29, 66^, resulting in additional impairment of the Smad1/5/9 pathway and inhibition of its protective antifibrotic role. Moreover, the silencing of Smad1 in NA HBFs by siRNA mediated the signal transfer from the antifibrotic TGF-β/Smad1/5/9 to the profibrotic TGF-β/Smad2/3 pathway and stimulated FMT in vitro.

BMP7 is known as a cytokine that can antagonize the TGF-β_1_-dependent fibrogenic activity of mouse pulmonary myofibroblastic cells^62^. This cytokine is also a main stimulator of the Smad1/5/9 pathway^43^. We showed that canonical BMP7 stimulation of the Smad1/5/9 pathway dysregulates α-SMA synthesis in both untreated and TGF-β_1_-treated HBFs from healthy donors. Similar results have been obtained by Pegorier et al. and Midgley et al. on normal human lung fibroblasts cultured in vitro in combination with TGF-β_1_^67, 68^. In our model, BMP7 stimulation of the Smad1/5/9 pathway reduced the efficiency of TGF-β_1_-induced FMT in AS HBFs by decreasing the percentage of myofibroblasts in HBF populations. Surprisingly, cotreatment of AS HBFs with TGF-β_1_ and BMP7 did not decrease α-SMA production; however, we observed disruption of α-SMA incorporation into actin stress fibres^16, 38^ and a consequent reduction in the formation of myofibroblasts. It is well documented that myofibroblast formation in a variety of tissues, including human bronchi, demands not only humoral factors (e.g. TGF-β) but also mechanical stimuli (e.g. appropriate cell stiffness and/or the interaction of cells with ECM proteins)^4^. Previously we have shown that HBFs from asthmatics form an increased number of thick stress fibres accompanied by enlarged focal adhesions which correlate with high elastic modulus of asthmatic HBFs and their increased efficiency to the TGF-β-induced FMT^14^. Taken together, our results demonstrate that BMP-7 (without affecting the level of α-SMA) induces changes in the actin cytoskeleton architecture in HBFs causing decreased cell stiffness, which correlate with the reduced FMT in BMP-7-treated HBF populations. These findings may be related to the previously shown impairment of the TGF-β/Smad1/5/9 pathway in AS HBFs. This impairment may be a reason for the dysregulation of the Smad1/5/9 pathway by upstream stimulation by BMP7 and TGF-β_1_ (Fig. 7). Additionally, a recent report showed that in a mouse model of asthma, the balance between TGF-β_1_ and BMP7 could be used to predict the intensity of lung fibrosis^61^. Moreover, the levels of the TGF-β_1_ and TGF-β receptors, unlike the level of BMP7 and its receptors, were higher in the bronchi of asthmatics than the non-asthmatic controls^61, 65^. The results from multiple fibrotic disease models demonstrated that BMP7 expression is downregulated during tissue fibrosis and that treatment with BMP7 prevented fibrotic changes^36, 46, 61^. Despite the promising reports from various animal studies, exogenous BMP7 has not yet been approved for use in human fibrotic diseases or even in preclinical studies, probably because BMP7 is costly and its clinical use is limited due to the supraphysiological doses required for therapeutic efficacy, which cause severe side effects. BMP7 is also likely rapidly cleared from the blood^49^. Given the above results, we decided to search for small molecule activators of BMP signalling. Based on the results of Vrijens et al., we chose ISL as a small activator of Smad1/5/9 signalling^45^. We demonstrated for the first time that in our in vitro model, ISL at non-cytotoxic and non-cytostatic concentrations notably attenuated the TGF-β_1_-induced phenotypic transition of AS HBFs into myofibroblasts through Smad1/5/9 pathway stimulation (Fig. 7). As we noted previously, the experimental model used in this study mimics the basic properties of HBFs during asthma and the chronic inflammation crucial for this disease. Thus, our results indicated that this agent may attenuate in vivo airway wall remodelling via a TGF-β desensitizing effect on HBFs. Earlier studies showed that ISL decreased the levels of IL-4 and IL-5 in allergic asthma, significantly improving the function of the lungs^69, 70^, and it may relax guinea-pig trachea through multiple intracellular actions^71^. ISL supplementation reduced high fat diet (HFD)-induced adipose tissue fibrosis and the expression of fibrosis-related genes in mice^72^. A recent report showed that ISL can also attenuate monocrotaline-induced pulmonary hypertension via its anti-inflammatory and antiproliferative actions in rats^73^. Thus, our and other results suggest that ISL is a strong candidate for antifibrotic therapy in asthma.

Taken together, our data shed light on FMT signalling in bronchial asthma when the disordered balance between the Smad2/3 and Smad1/5/9 pathways is considered. Based on our results, we strongly suggest that enhanced asthma-related FMT (the key event of subepithelial fibrosis in the bronchial wall of asthmatic patients) depends on TGF-β/Smad1/5/(8)9 pathway impairment (Fig. 7). In connection with the above findings, the usage of substances stimulating this antifibrotic pathway (such as ISL) may prove to be an effective way to suppress FMT and subepithelial fibrosis in asthma, but the exact molecular mechanism of the phenomenon reported here requires further studies.

## Materials and methods

### Patient characteristics

For the study, 10 patients with asthma severity 3–5 (according to the Global Initiative for Asthma (GINA) classification) and 7 control subjects were included. All patients were treated in the Department of Medicine of the Jagiellonian University Medical College and remained in stable clinical conditions. The control subjects were selected from the group of patients who were referred for bronchoscopy due to prolonged cough. These patients had previous thorough, extensive diagnostics, during which other diseases causing cough were excluded by means of anamnesis and additional tests (imaging, lung function tests, histopathological examination of bronchial samples, allergic tests, microbiological tests). They were finally diagnosed with post-infectious or idiopathic cough. All participants have never smoked. COPD was excluded by the history and the correct result of spirometry, including FEV_1_/FVC ratio after bronchodilator > 0.7. Therefore, it did not affect the results.

Detailed characteristics of asthma patients and control subjects are presented in Table 1.

**Table 1:** Patient characteristics.

	Asthmatic patients (n = 10)	Control subjects (n = 7)
Sex (Female/Male)	6/4	4/3
Age (years), mean ± SD	42.8 ± 15.2	56.8 ± 9.5
FEV_1_% predicted, mean ± SD	67.3 ± 22.5	97.17 ± 11.1
FEV_1_/FVC ratio before bronchodilator, mean ± SD	0.65 ± 0.6	0.83 ± 0.7
FEV_1_/FVC ratio after bronchodilator, mean ± SD	0.75 ± 0.5	0.85 ± 0.4
Asthma duration (years), mean ± SD	17.0 ± 13.8	NA
Treatment with ICS, n (%)	7 (70)	NA
ICS (µg/day)^a^, mean ± SD	642.9 ± 349.3	NA
Treatment with OCS, n (%)	3 (30)	NA
OCS (µg/day)^b^, mean ± SD	8.7 ± 7.0	NA

ICS, inhaled corticosteroids; OCS, oral corticosteroids; NA, non-applicable.

^a^Maintenance dose, fluticasone propionate equivalent.

^b^Maintenance dose, methylprednisolone equivalent.

Although the studied groups of donors differ in the mean age, in our opinion, a younger age of asthma patients in comparison to control subjects has no effect on the study outcomes. Age differences are mainly due to the fact that it was difficult to recruit theoretically “healthy” subjects for the control group and to collect from them samples from the lower respiratory tract by bronchoscopy. Therefore, we recruited all suitable subjects regardless their age.

The study was approved by the Jagiellonian University Ethics Committee (Decision No. 122.6120.16.2016). All patients and control subjects gave written informed consent to participate in the study.

All methods were performed in accordance with the relevant guidelines and regulations.

### Isolation and culture of primary HBFs

Primary HBFs were established from the explants of bronchial biopsies obtained from patients during bronchoscopy using a fiberoptic bronchoscope (Olympus). Then, the explants were washed with cold phosphate buffered saline (PBS, Corning) and transferred to fibroblast growth medium (FGM; Fibroblast Basal Medium containing 2% FBS and supplements from the FGM-2 Bullet Kit; Lonza) with collagenase IV (Worthington, USA; 1 mg/ml) at 37 °C. After 5–6 h of incubation with intensive mixing several times, the digested samples were centrifuged for 5 min at 300×*g*, transferred to Petri dishes and maximally extended using sterile needles. Primary cultures of HBFs were established in FGM for 3 weeks with FGM replacement every 48–72 h. Next, the cells were passaged and, after centrifugation (300×*g*; 5 min), suspended in complete Dulbecco’s modified Eagle’s medium containing high glucose (DMEM, Sigma-Aldrich, St. Louis, MO, USA) supplemented with 10% foetal bovine serum (FBS, GibcoTM, Thermo Fisher Scientific) and a penicillin/streptomycin cocktail (P4333; Sigma-Aldrich, St. Louis, MO, USA). Cells were cultured in standard conditions (37 °C and 5% CO2) to 80–85% confluence and used for experiments between the 5th and 15th passages. We used cells from new isolations (fresh cells) and cells that were deposited in the cell bank (frozen cells). Cells from similar passages (± 1 passage) as well as fresh cells or frozen cells from both studied groups were used for each experiment comparing HBFs AS and NA. The phenotype of established primary cultures of HBFs was verified by immunofluorescence staining of α-SMA, vimentin and desmin^14^. All cells expressed vimentin-positive staining, whereas desmin expression was not observed. In HBF populations derived from asthmatic patients, α-SMA–positive staining was observed in ca. 5% cells. For each experiment, cells were seeded at a density of 5000 cells/cm^2^, cultured in complete medium for 24 h, and then cultured in serum-free medium—DMEM containing 0.1% bovine serum albumin (BSA, Sigma-Aldrich, St. Louis, MO, USA) in the absence or presence of human recombinant TGF-β1 (5 ng/ml; BD Bioscience; dissolved in a solution of 1 mg/ml BSA/PBS) and/or BMP7 (100 ng/ml Sigma-Aldrich, St. Louis, MO, USA) and/or ISL (25 μM; Sigma-Aldrich, St. Louis, MO, USA).

### Cytotoxicity and proliferation assays

For the determination of the cytotoxic/cytostatic effect of ISL on HBFs we seeded cells at a concentration of 2.5 × 10^4^ cells/cm^2^ in culture medium into microplates (96 wells, flat bottom; VWR). 24 h after seeding, we added different concentrations of ISL (12.5 μM, 25 μM, 50 μM, 75 μM, 100 μM). After the incubation period (24 h, 72 h and 4 days), we performed the MTT assay (due to manufacturer protocol) for determination of cytotoxicity and Crystal Violet assay for determination of cells’ proliferation. The absorbance of the samples was measured using a microplate reader (Multiscan FC; Thermo Fisher Scientific). The wavelength to measure absorbance of the formazan product was 570 nm and for crystal violet was 540 nm. IC50 was calculated for each time of incubation with ISL.

### Gene silencing

Silencing of Smad1 expression in HBFs was conducted in antibiotic-free medium supplemented with 10% FBS using Lipofectamine™2000 according to the manufacturer’s protocol as a carrier for small interfering RNA (10 µM solution of siRNA-Smad1 duplex; sc-29483; Control siRNA-A; sc-37007; Santa Cruz Biotechnology). After 4 h of incubation, the medium was replaced with serum-free DMEM with 0.1% BSA and antibiotics. The transfected HBFs (silencing efficiency about 60%; Fig. S1C) were used for further experiments.

### Immunofluorescence studies

HBFs were cultured for different periods (1 min–2 h for p-Smads; 7 days for α-SMA staining) in serum-free medium in the presence or absence of TGF-β_1_ and then fixed with 3.7% formaldehyde/PBS. The following antibodies were used: anti-Smads/p-Smads from Smad1/5/9 (#12656) and Smad2/3 (#12747) Antibody Sampler Kits (Cell Signaling; all: 1:100). Stress fibres containing α-SMA were visualized using mouse monoclonal IgG against α-SMA (Sigma-Aldrich, St. Louis, MO, USA) with appropriate secondary antibodies (goat-anti mouse IgG conjugated with Alexa Fluor 488). Samples were counterstained by phalloidin conjugated with Alexa Fluor 546 (Life Technologies, Thermo Fisher Scientific). Some samples were counterstained with Hoechst 33258 (1 µg/ml, Sigma-Aldrich, St. Louis, MO, USA). The images were acquired using a Leica DMI6000B inverted microscope (Leica Microsystems, Wetzlar, Germany) equipped with LAS-X software for image processing. Smad signalling pathway activation is presented as a percentage of cells with strong signals of the phosphorylated forms of Smads from the nuclear area. In turn, the effectiveness of FMT was determined by the percentage of cells with α-SMA-positive microfilaments.

Quantification of the obtained results was performed using plot profiles perpendicular to bundles of stress fibres with quantification of the signal for particular spikes or fluorescence intensity of the nuclear areas (for Smad activity). Fiji ImageJ 1.51 s was used to measure these signals. Finally, the averaged fluorescence signal of the analysed p-Smads (for at least 100 cell nuclei) in relation to the averaged DNA-specific fluorescence intensity from the surface of proper cell nuclei, was compared between the conditions. Cytofluorimetric analyses of p-Smad levels were performed using the same excitation/exposure settings^16, 38^.

### Immunoblots

Preparation of protein supernatant from cultured cells was described previously^16, 38^. Samples containing 20 µg of proteins were electrophoresed on a 10% SDS–polyacrylamide gel and transferred to PVDF membranes (Bio-Rad) according to a previously described protocol^16, 38^.

Next, the membranes were incubated overnight at 4 °C with primary antibodies against Smad proteins from the Smad2/3 Antibody Sampler Kit and the Smad1/5/9 antibody Sampler Kit, mouse monoclonal IgG against α-SMA, mouse monoclonal IgG against β-tubulin, mouse monoclonal IgG against β-actin, mouse monoclonal IgG against vinculin and mouse monoclonal IgM against glyceraldehyde 3 phosphate dehydrogenase (GAPDH) (All: Sigma-Aldrich, St. Louis, MO, USA; 1:1000), diluted in 1% BSA/PBS. After three washes with Tris-buffered saline with Tween 20 (TBST), the membranes were exposed to horseradish peroxidase-conjugated anti-mouse or anti-rabbit IgG (all: 1:3000, Life Technologies). Band detection was performed using Luminata Crescendo Western HRP Substrate (Merck Millipore), and the chemiluminescence imaging system ChemiDoc XRS + (Bio-Rad) was used. Band intensities were quantified using Fiji ImageJ 1.51 s freeware.

### Cell-based enzyme-linked immunosorbent assay (in-cell-ELISA)

HBFs were grown in 96-well plates in serum-free medium alone or in combination with human recombinant TGF-β_1_ (5 ng/ml) for 7 days. An *in cell* ELISA protocol^38^ was performed using antibodies against α-SMA (the same as used in the immunofluorescence analyses and Western blot) or fibronectin (1:2000 in 1% BSA/PBS). The results are presented as an absorbance value (450 nm; Microplate Reader, Thermo Scientific, Multiskan FC) corresponding to the relative amount of protein levels.

### Real-time PCR

Isolation of mRNA from HBFs was performed using the GeneMATRIX Universal RNA/miRNA Purification Kit (EURx, Gdańsk, Poland) according to the manufacturer’s protocol. The concentration of isolated mRNA was measured in a NanoDrop spectrophotometer (Implen) at OD260/280 nm. Two thousand nanograms of extracted mRNA, the NG dART RT-PCR Kit (EURx) and C1000 Touch Thermal Cycler (Bio-Rad) were used for cDNA synthesis. Gene expression was measured with SYBR Green PCR Master Mix (Applied Biosystems) and specific primer sets (described in Table 2; all from Genomed, Warszawa, Poland) using the 7500 Fast System (Applied Biosystems). Relative amounts of genes were estimated using the quantification threshold value recalculated against *GAPDH* transcripts by the ^∆^CT method [^∆^CT refers to CT_(tested gene)_-CT_(GAPDH)_] and are presented as a 2^-ΔCt mean^ value.

**Table 2:** Primers used for quantitative PCR.

Gene name	Sequence 5′–3'	
	Forward	Reverse
ACTA2 (α-SMA)	CTGTTCCAGCCATCCTTCAT	CCGTGATCTCCTTCTGCATT
Smad1	ACCTGCTTACCTGCCTCCTG	CATAAGCAACCGCCTGAACA
Smad2	CGTCCATCTTGCCATTCACG	CTCAAGCTCATCTAATCGTCCTG
Smad3	GCGTGCGGCTCTACTACATC	GCACATTCGGGTCAACTGGTA
Smad5	CTGGGATTACAGGACTTGACC	AAGTTCCAATTAAAAAGGGAGGA
TGF-β_1_	AGGGCTACCATGCCAACTTCT	CCGGGTTATGCTGGTTGTACA
GAPDH	GAAGGTGAAGGTCGGAGT	GAAGATGGTGATGGGATTTC

### Statistical analysis

The statistical significances were determined using the non-parametric Mann–Whitney test (the comparison between groups HBFs NA and HBFs AS) or the t-test (the comparison in one group between two different experimental conditions) due to the small number of groups and a lack of normal distribution of data. For the data with normal distribution of data (number of measurements > 50), statistical significance was tested using one-way ANOVA with the Bonferroni multiple comparison post hoc test. The differences were considered to be statistically significant at probability levels of *p < 0.05%, **p < 0.01%, ***p < 0.001%. Each parameter was calculated as the mean (± SEM).

## Supplementary information

Supplementary Information.

## References

[1] Grainge CL (2011). Effect of bronchoconstriction on airway remodeling in asthma. N. Engl. J. Med..

[2] Baldwin L, Roche WR (2002). Does remodelling of the airway wall precede asthma?. Paediatr. Respir. Rev..

[3] Martin JG, Verma N (2012). Mechanisms of airway remodeling in asthma. Drug Discov. Today Dis. Mech..

[4] Michalik M (2018). Fibroblast-to-myofibroblast transition in bronchial asthma. Cell. Mol. Life Sci..

[5] Fehrenbach H, Wagner C, Wegmann M (2017). Airway remodeling in asthma: what really matters. Cell Tissue Res..

[6] Duffield JS, Lupher M, Thannickal VJ, Wynn TA (2013). Host responses in tissue repair and fibrosis. Annu. Rev. Pathol. Mech. Dis..

[7] Hinz B (2007). The myofibroblast: one function, multiple origins. Am. J. Pathol..

[8] Kramann R, DiRocco DP, Humphreys BD (2013). Understanding the origin, activation and regulation of matrix-producing myofibroblasts for treatment of fibrotic disease. J. Pathol..

[9] Di Carlo SE, Peduto L (2018). The perivascular origin of pathological fibroblasts. J. Clin. Investig..

[10] Darby IA, Zakuan N, Billet F, Desmoulière A (2016). The myofibroblast, a key cell in normal and pathological tissue repair. Cell. Mol. Life Sci..

[11] El Agha E (2017). Mesenchymal stem cells in fibrotic disease. Cell Stem Cell.

[12] Michalik M (2009). Asthmatic bronchial fibroblasts demonstrate enhanced potential to differentiate into myofibroblasts in culture. Med. Sci. Monit..

[13] Sugiura H (2007). Cultured lung fibroblasts from ovalbumin-challenged ‘asthmatic’ mice differ functionally from normal. Am. J. Respir. Cell Mol. Biol..

[14] Sarna M (2015). Undifferentiated bronchial fibroblasts derived from asthmatic patients display higher elastic modulus than their non-asthmatic counterparts. PLoS ONE.

[15] Michalik M (2011). Transition of asthmatic bronchial fibroblasts to myofibroblasts is inhibited by cell-cell contacts. Respir. Med..

[16] Paw M (2017). Connexin43 controls the myofibroblastic differentiation of bronchial fibroblasts from patients with asthma. Am. J. Respir. Cell Mol. Biol..

[17] Wójcik-Pszczoła K (2018). Connective tissue growth factor regulates transition of primary bronchial fibroblasts to myofibroblasts in asthmatic subjects. Cytokine.

[18] Wójcik KA, Koczurkiewicz P, Michalik M, Sanak M (2012). Transforming growth factor-β1-induced expression of connective tissue growth factor is enhanced in bronchial fibroblasts derived from asthmatic patients. Pol. Arch. Med. Wewn..

[19] Moffatt MF (2010). Europe PMC Funders Group Europe PMC funders author manuscripts a large-scale, consortium-based genomewide association study of asthma. N. Engl. J. Med..

[20] Yao Y, Chang W, He L, Jin Y, Li C (2016). An updated meta-analysis of transforming growth factor-β1 gene: three well-characterized polymorphisms with asthma. Hum. Immunol..

[21] Duvernelle C, Freund V, Frossard N (2003). Transforming growth factor-β and its role in asthma. Pulm. Pharmacol. Ther..

[22] Halwani R, Al-Muhsen S, Al-Jahdali H, Hamid Q (2011). Role of transforming growth factor-β in airway remodeling in asthma. Am. J. Respir. Cell Mol. Biol..

[23] Redington AE (1997). Transforming growth factor-β1 in asthma: measurement in bronchoalveolar lavage fluid. Am. J. Respir. Crit. Care Med..

[24] Graham H, Peng C (2006). Activin receptor-like kinases: structure, function and clinical implications. Endocr. Metab. Immune Disord. Drug Targets.

[25] Moustakas A, Heldin C-H (2005). Non-Smad TGF-β signals. J. Cell Sci..

[26] Budi EH, Duan D, Derynck R (2017). Transforming growth factor-β receptors and smads: regulatory complexity and functional versatility. Trends Cell Biol..

[27] Derynck R, Zhang YE (2003). Smad-dependent and Smad-independent pathways in TGF-β family signalling. Nature.

[28] Massagué J, Seoane J, Wotton D (2005). Smad transcription factors. Genes Dev..

[29] Zhang Y, Chang C, Gehling DJ, Hemmati-Brivanlou A, Derynck R (2001). Regulation of Smad degradation and activity by Smurf2, an E3 ubiquitin ligase. Proc. Natl. Acad. Sci. USA.

[30] Pohlers D (2009). TGF-β and fibrosis in different organs—molecular pathway imprints. Biochim. Biophys. Acta.

[31] Xu F, Liu C, Zhou D, Zhang L (2016). TGF-β/SMAD pathway and its regulation in hepatic fibrosis. J. Histochem. Cytochem..

[32] Biernacka A, Dobaczewski M, Frangogiannis NG (2011). TGF-β signaling in fibrosis. Growth Factors.

[33] Wang W, Koka V, Lan HY (2005). Transforming growth factor-β and Smad signalling in kidney diseases. Nephrology.

[34] Muñoz-Félix JM, González-Núñez M, Martínez-Salgado C, López-Novoa JM (2015). TGF-β/BMP proteins as therapeutic targets in renal fibrosis. Where have we arrived after 25 years of trials and tribulations?. Pharmacol. Ther..

[35] McVicker BL, Bennett RG (2017). Novel anti-fibrotic therapies. Front. Pharmacol..

[36] Michalik M (2012). Lithium attenuates TGF-β1-induced fibroblasts to myofibroblasts transition in bronchial fibroblasts derived from asthmatic patients. J. Allergy.

[37] Michalik M (2013). Lovastatin-induced decrease of intracellular cholesterol level attenuates fibroblast-to-myofibroblast transition in bronchial fibroblasts derived from asthmatic patients. Eur. J. Pharmacol..

[38] Paw M (2018). Fenofibrate reduces the asthma-related fibroblast-to-myofibroblast transition by TGF-Β/Smad2/3 signaling attenuation and connexin 43-dependent phenotype destabilization. Int. J. Mol. Sci..

[39] Muñoz-Félix JM, González-Núñez M, López-Novoa JM (2013). ALK1-Smad1/5 signaling pathway in fibrosis development: Friend or foe?. Cytokine Growth Factor Rev..

[40] Dexheimer V (2016). Differential expression of TGF-β superfamily members and role of Smad1/5/9-signalling in chondral versus endochondral chondrocyte differentiation. Sci. Rep..

[41] Jaiswal H, Dev DS, Kisku DR (2017). Rank order reduction based fast pattern matching algorithm. Gut.

[42] Weiskirchen R (2009). BMP-7 as antagonist of organ fibrosis. Front. Biosci..

[43] Miyazawa K, Shinozaki M, Hara T, Furuya T, Miyazono K (2002). Two major Smad pathways in TGF-β superfamily signalling. Genes Cells.

[44] Wang RN (2014). Bone morphogenetic protein (BMP) signaling in development and human diseases. Genes Dis..

[45] Vrijens K (2013). Identification of small molecule activators of BMP signaling. PLoS ONE.

[46] Breton J, Heydet D, Starrs LM, Veldre T, Ghildyal R (2018). Molecular changes during TGF β-mediated lung fibroblast-myofibroblast differentiation: implication for glucocorticoid resistance. Physiol. Rep..

[47] Kurundkar A, Thannickal VJ (2016). Redox mechanisms in age-related lung fibrosis. Redox Biol..

[48] Sampson N, Berger P, Zenzmaier C (2012). Therapeutic targeting of redox signaling in myofibroblast differentiation and age-related fibrotic disease. Oxid. Med. Cell. Longev..

[49] Walton KL, Johnson KE, Harrison CA (2017). Targeting TGF-β Mediated SMAD signaling for the prevention of fibrosis. Front. Pharmacol..

[50] Koćwin M, Jonakowski M, Przemęcka M, Panek M, Kuna P (2017). Selected bone morphogenetic proteins—the possibility of their use in the diagnostics and therapy of severe asthma. Adv. Respir. Med..

[51] Sagara H (2002). Activation of TGF-β/Smad2 signaling is associated with airway remodeling in asthma. J. Allergy Clin. Immunol..

[52] Lee HY (2017). Inhibitory effects of resveratrol on airway remodeling by transforming growth factor-β/smad signaling pathway in chronic asthma model. Allergy Asthma Immunol. Res..

[53] Qu ZH (2012). Inhibition airway remodeling and transforming growth factor-β1/Smad signaling pathway by astragalus extract in asthmatic mice. Int. J. Mol. Med..

[54] Torrego A, Hew M, Oates T, Sukkar M, Kian FC (2007). Expression and activation of TGF-β isoforms in acute allergen-induced remodelling in asthma. Thorax.

[55] Kariyawasam HH (2009). Activin and transforming growth factor-β signaling pathways are activated after allergen challenge in mild asthma. J. Allergy Clin. Immunol..

[56] Eap R, Jacques E, Semlali A, Plante S, Chakir J (2012). Cysteinyl leukotrienes regulate TGF-Β1 and collagen production by bronchial fibroblasts obtained from asthmatic subjects. Prostaglandins Leukot. Essent. Fat. Acids.

[57] Koczurkiewicz P (2016). Synergistic cytotoxic and anti-invasive effects of mitoxantrone and triterpene saponins from lysimachia ciliata on human prostate cancer cells. Planta Med..

[58] Wrighton KH, Lin X, Yu PB, Feng XH (2009). Transforming growth factor β can stimulate Smad1 phosphorylation independently of bone morphogenic protein receptors. J. Biol. Chem..

[59] Zhang H, Du L, Zhong Y, Flanders KC, Roberts JD (2017). Transforming growth factor-β stimulates Smad1/5 signaling in pulmonary artery smooth muscle cells and fibroblasts of the newborn mouse through ALK1. Am. J. Physiol..

[60] Meng X-M, Chung ACK, Lan HY (2013). Role of the TGF-β/BMP-7/Smad pathways in renal diseases. Clin. Sci..

[61] Stumm CL (2014). Lung remodeling in a mouse model of asthma involves a balance between TGF-β1 and BMP-7. PLoS ONE.

[62] Izumi N (2006). BMP-7 opposes TGF-β1-mediated collagen induction in mouse pulmonary myofibroblasts through Id2. Am. J. Physiol. Cell. Mol. Physiol..

[63] Liang D (2016). BMP-7 attenuated silica-induced pulmonary fibrosis through modulation of the balance between TGF-β/Smad and BMP-7/Smad signaling pathway. Chem. Biol. Interact..

[64] Chen X, Xu J, Jiang B, Liu D (2016). Bone morphogenetic protein-7 antagonizes myocardial fibrosis induced by atrial fibrillation by restraining transforming growth factor-β (TGF-β)/Smads signaling. Med. Sci. Monit..

[65] Kariyawasam HH (2008). Basal expression of bone morphogenetic protein receptor is reduced in mild asthma. Am. J. Respir. Crit. Care Med..

[66] Tang LY (2011). Ablation of Smurf2 reveals an inhibition in TGF-β signalling through multiple mono-ubiquitination of Smad3. EMBO J..

[67] Pegorier S, Campbell GA, Kay AB, Lloyd CM (2010). Bone morphogenetic protein (BMP)-4 and BMP-7 regulate differentially transforming growth factor (TGF)-β1 in normal human lung fibroblasts (NHLF). Respir. Res..

[68] Midgley AC (2015). Hyaluronan regulates bone morphogenetic protein-7-dependent prevention and reversal of myofibroblast phenotype. J. Biol. Chem..

[69] Yang N (2013). Glycyrrhiza uralensis flavonoids present in anti-asthma formula, ASHMI^TM^, inhibit memory Th2 responses in vitro and in vivo. Phyther. Res..

[70] Wen MC (2005). Efficacy and tolerability of antiasthma herbal medicine intervention in adult patients with moderate-severe allergic asthma. J. Allergy Clin. Immunol..

[71] Liu B, Yang J, Wen Q, Li Y (2008). Isoliquiritigenin, a flavonoid from licorice, relaxes guinea-pig tracheal smooth muscle in vitro and in vivo: Role of cGMP/PKG pathway. Eur. J. Pharmacol..

[72] Watanabe Y (2016). Isoliquiritigenin attenuates adipose tissue inflammation in vitro and adipose tissue fibrosis through inhibition of innate immune responses in mice. Sci. Rep..

[73] Jin H (2019). Isoliquiritigenin attenuates monocrotaline-induced pulmonary hypertension via inhibition of the inflammatory response and PASMCs proliferation. Evid.-Based Complement. Altern. Med..

