# Supplementary material for: Enhanced asthma-related fibroblast to myofibroblast transition is the result of profibrotic TGF-β/Smad2/3 pathway intensification and antifibrotic TGF-β/Smad1/5/(8)9 pathway impairment
Source: Sci Rep. 2020 Oct 5;10:16492. doi: 10.1038/s41598-020-73473-7 (PMC7536388; doi:10.1038/s41598-020-73473-7)
Supplement: Supplementary file 1 — Supplementary Information. [file 41598_2020_73473_MOESM1_ESM.docx]

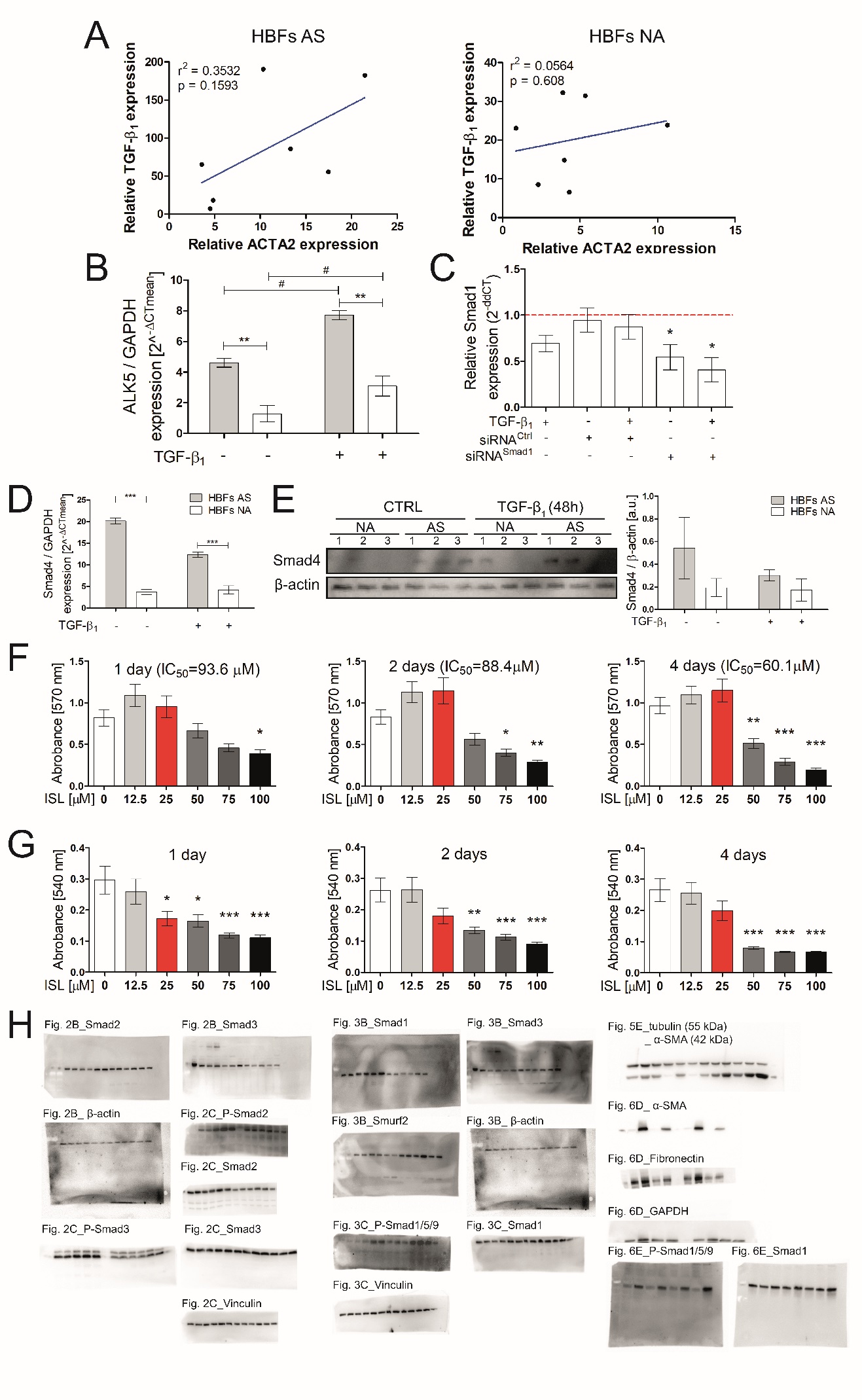


**Figure S1.**

**(A)** HBFs (AS: n=7; NA: n=7) were cultured in serum-free conditions in the presence of TGF-β_1_ (5ng/ml) for 24 h. RT-qPCR analyses of α-smooth muscle actin (*ACTA2*) and *TGF-β_1_* expression were performed. Linear regression has been plotted.

**(B)** Cells were cultured in a serum-free medium supplemented with TGF-β_1_ (5 ng/mL) for 24 h. Then, the mRNA was isolated, and transcripts of ALK5 and Smad4 were analysed using RT-qPCR.

**(C)** HBFs (n=4) cultured in serum-free medium were transfected with siRNA-Smad1 or control-siRNA were treated with TGF-β_1_ (5 ng/ml) for 24 h. Next the RT-qPCR analyse of the level of Smad1 silencing was performed. Red line corresponds to control cells.

**(D)** HBFs were cultured in a serum-free medium with or without TGF-β_1_ (5 ng/mL) for 24 h. RT-qPCR analyses of Smad 4 expression were performed.

**(E)** Cells were cultured in serum-free medium supplemented with TGF-β_1_ (5 ng/mL) for 48 h. Smad4 was detected using immunoblotting.

**(F)** HBFs (n=8) were cultured in microplates with or without ISL (12.5 μM, 25 μM, 50 μM, 75 μM, 100 μM) for 24 h, 48 h and 4 days. MTT assay were performed and absorbance of formazan product was measured (570 nm). IC50 was calculated for each time of incubation.

**(G)** HBFs (n=8) were cultured in microplates with or without ISL (12.5 μM, 25 μM, 50 μM, 75 μM, 100 μM) for 24 h, 48 h and 4 days. Crystal Violet assay used to determination of cell proliferation were performed and absorbance of violet was measured (540 nm).

**(H)** The original, unprocessed versions of immunoblot membranes from Fig. 2 up to Fig.5
